# The impact of progredient vessel and tissue stiffening for the development of metabolic syndrome

**DOI:** 10.1007/s00424-022-02749-w

**Published:** 2022-09-23

**Authors:** Clemens Loracher, Bruno Märkl, Alois Loracher

**Affiliations:** General Pathology and Molecular Diagnostics, Medical Faculty, University Augsburg, Stenglinstrasse 2, 86156 Augsburg, Germany

**Keywords:** Metabolic syndrome, Arteriosclerosis, Stiffness, Mechanosensitive cation channels

## Abstract

Established risk factors for the metabolic syndrome as diabetes and arterial hypertension are believed to be the cause of arteriosclerosis and subsequently following diseases like coronary heart disease, apoplexy, or chronic renal failure. Based on broad evidence from the already available experimental literature and clinical experience, an alternative hypothesis is presented that puts an increased vessel and organ stiffness to the beginning of the pathophysiological scenario. The stiffness itself is caused by a persistent activation of mechano-sensitive cation channels like the epithelial/endothelial sodium channel. A further enhancement takes place by proteins like JACD and RhoA coupled phospholipase C coupled G-protein receptors and integrins. A self-enhancing positive feedback loop by activation of YAP/TAZ signaling is a further central pillar of this theory. Further investigations are necessary to verify this hypothesis. If this hypothesis could be confirmed fundamental changes regarding the pharmacologic therapy of the diseases that are currently summarizes as metabolic syndrome would be the consequence.

## Introduction

Sedentary lifestyle, hypercaloric diet rich in fat and carbohydrates, excessive salt supply combined with nicotine abuse and disturbed day-night rhythms lead in “western” countries to an epidemic-like increase in dyslipidemia, insulin-resistant obesity/type 2 diabetes mellitus (T2D), and arterial hypertension. Occurring frequently together, these entities are also uniformly subsumed as metabolic syndrome (MS) without an explanatory pathophysiology for this coincidence [4].

The components of the metabolic syndrome (risk factors) are considered to be the cause of the development of arteriosclerosis and their secondary diseases (e.g., coronary heart disease/myocardial infarction, apoplexy, arterial occlusive disease, sclerotic renal insufficiency, and non-alcoholic steatohepatitis (NASH)). Implied herein is a direct proportionality between the magnitude of the risk factors and the severity of arteriosclerotic disease.

However clinical and biologic findings point to something different:

Comprehensive long-term data from the Framingham study show that contrary to the common pathophysiological concept, an increase in arterial stiffness (corresponding to arteriosclerosis) precedes the development of arterial hypertension and is not its consequence but its cause [5]. Substantiating and expanding the Framingham data, Agbaje et al. prove increased arterial stiffness and wall thickness to precede hypertension, insulin resistance, and dyslipidemia [1, 2]. Despite comparable hypertension, patients with primary aldosteronism show in contrast to patients with essential hypertension a significantly higher mortality and cardiovascular/metabolic complication rate. The aldosterone effect is mediated to a high extent by the nearly ubiquitarily expressed epithelial/endothelial sodium channel (ENaC) (e.g. distal renal tubes, endothelium, vascular smooth muscles, dendritic cells, specific neurons). That expressed epithelial/endothelial sodium channel (ENaC).

Epidemiological studies of insulin therapy in T2D — which stimulates ENaC — show increased risk of cardiovascular disease and death [6]. Pathophysiology-based sub phenotyping of individuals at elevated risk for T2D identifies a group of persons with increased risk of complications and death without rapid progression to manifest diabetes [9].

Patients with NASH show independent of hypertension and an increase in diastolic heart failure, atrial fibrillation, nephro-sclerotic renal insufficiency, coronary artery disease, and degenerative aortic stenosis, which cannot be explained by the concomitant metabolic changes, and thus indicate a common, yet unclarified, pathogenetic mechanism.

## Stiffness as cause but not result of arteriosclerosis

**Fig. 1:**
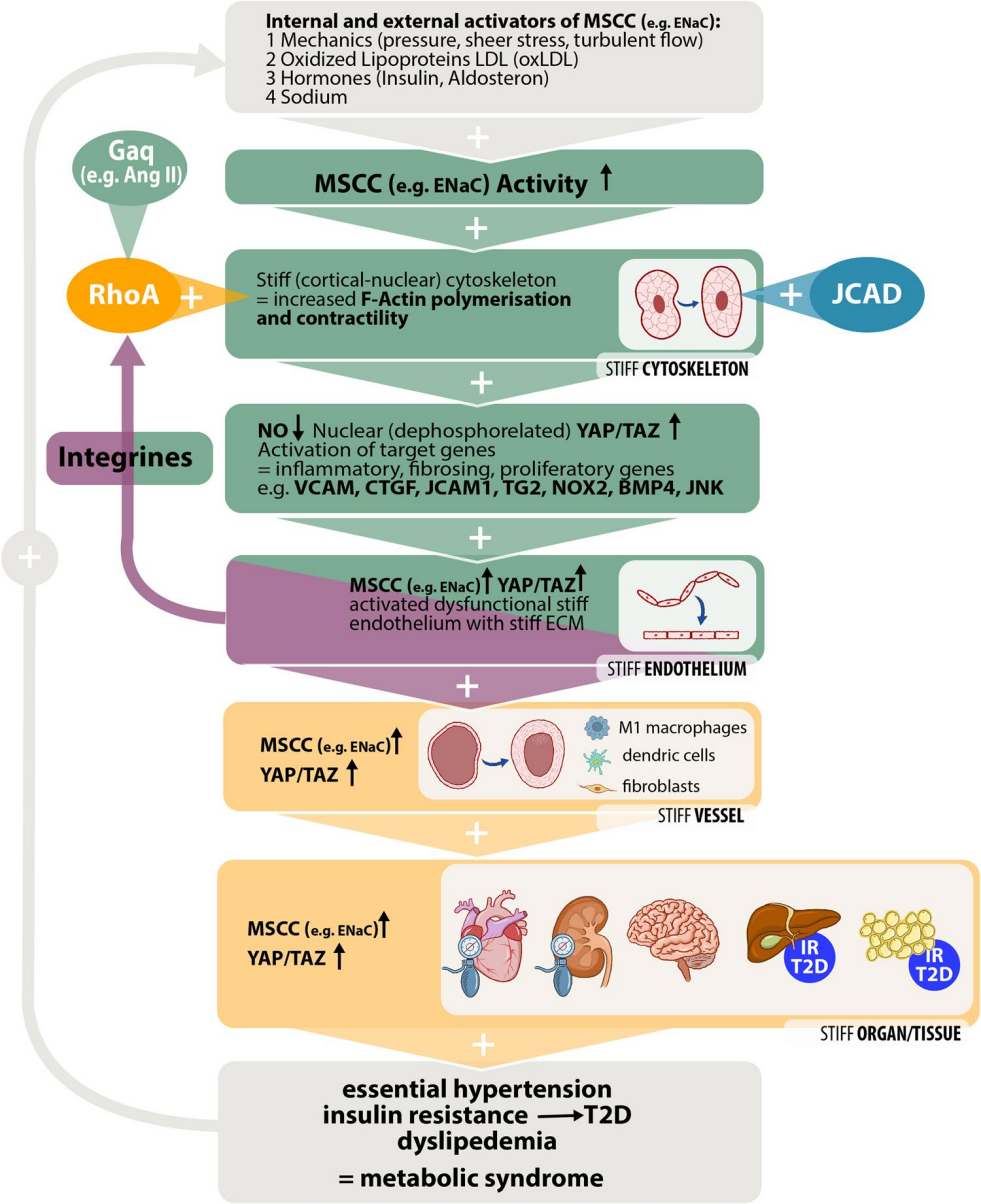
Feedback loops stiff tissue/metabolic syndrome. Persistent mechanosensitive cation channel (MSCC; paradigmatically represented by ENaC) activation (external mainly by “western diet,” sedentary lifestyle, high sodium intake) leads to endothelial cell stiffening by stimulation of F-actin polymerization and contractility. Cytoskeletal stiffness is further enhanced by junctional proteins like JACD and RhoA coupled phospholipase C coupled G-protein (Gαq) (e.g., angiotensin II) receptors and integrins. Elevated cytoskeletal stiffness reduces NO and induces nuclear translocation of dephosphorylated YAP/TAZ and activation of their target genes. Their activity culminates in the pathophysiological picture of activated/dysfunctional (stiff) endothelium with rigid ECM. High ECM stiffness in turn activates via integrins/RhoA further cytoskeletal rigor, thus closing a positive feedback loop. Stiff endothelium-induced and maintained by enhanced activity of ENaC and YAP/TAZ leads via inflammatory and fibrosing mediators to stiff vessel phenotype (characterized by elevated ENaC and YAP/TAZ action in multiple cell types). Reciprocal mechano-metabolic coupling of vessels and organs/tissues induces stiff organ/tissue phenotype, again showing elevated ENaC and YAP/TAZ effects. In the wake of organ/tissue stiffening, the components of metabolic syndrome emerge, closing by further endothelial stiffening another positive feedback loop

According to clinical and multiple experimental studies, the mechanosensitive ENaC plays a key role in the pathogenesis of arteriosclerotic vascular disease. (ENaC stands paradigmatically “pars pro toto” for a large group of mechanosensitive, cell membrane–located cation channels (e.g., PIEZO1/2, TRPC1/6) functionally linked to the cytoskeleton and co-regulated by the extracellular glycocalyx) Its expression and activation are amplified by mechanical factors (pressure, shear forces, turbulent flow), high sodium, oxidized LDL (western diet), and hormones like insulin and aldosterone. Persistent ENaC activation leads to endothelial cell stiffening by stimulation of cytoskeletal F-actin polymerization and contractility. Stiffness is further enhanced by junctional proteins like JACD and dominantly by RhoA coupled Gαq (e.g., angiotensin II) receptors and integrins [7]. Elevated cytoskeletal stiffness reduces NO synthetase activity and induces nuclear translocation of dephosphorylated YAP/TAZ and consecutive activation of their target genes — potent mediators of proliferation, dedifferentiation, inflammation, and fibrosis [10]. Their activity culminates in the pathophysiological picture of activated/dysfunctional (stiff) endothelium with disrupted inhibitory glycocalyx function on ENaC, integrins, and Gaq-proteins resulting in a rigid extracellular matrix (ECM) [3]. High ECM stiffness in turn activates via integrins/Ras homologue A (RhoA) further cytoskeletal rigor, thus closing a positive feedback loop (Fig. 1). Stiff endothelium induced and maintained by enhanced activity of ENaC and yes associated protein (YAP)/transcriptional coactivator with PDZ binding motif (TAZ) leads via inflammatory and fibrosing mediators to stiff vessel phenotype, characterized by elevated ENaC activity and YAP/TAZ action in multiple cell types (e.g., dendritic cells and smooth muscle cells). Reciprocal mechano-metabolic coupling of vessels and organs/tissues induces stiff organ/tissue phenotype, again showing elevated ENaC and YAP/TAZ effects [8]. In the wake of organ/tissue stiffening, the components of metabolic syndrome emerge:

The stiff tissue phenotype of adipose tissue promotes macrophage prominent perivascular fibrosis with dedifferentiated, dysfunctional adipocytes. This results in reduced fat storage capacity and, by selective impairment of components of insulin action, to lipolysis (Other insulin signaling cascades remain intact and are overactive due to coexistent hyperinsulinemia). Consecutively, hepatic, glycerol-triggered glucogenesis is induced, manifesting the typical insulin resistance syndrome observed in MS.

The stiff liver phenotype (NASH), showing close coupling with stiff adipose tissue, is characterized by ectopic fat storage and increased insulin-independent lipo/glucogenesis, functionally resulting in pronounced dyslipidemia and insulin-resistant hyperglycemia, which can lead to the development of T2D.

The stiff organ phenotype leads in the brain to glymphatic dysfunction following neurodegeneration (e.g. Alzheimer’s disease, Parkinson’s disease) and to gliosis of the mediobasal hypothalamus causing leptin-resistant dyslipidemia and T2D. Furthermore, increased ENaC/endogenous ouabain activity leads to a higher CNS sympathetic tone and development of secondary hypertension.

The renal stiff organ phenotype is reflected in glomerulosclerosis-induced renal insufficiency and secondary hypertension.

The atherosclerotic generated increase in arterial stiffness and baroreceptor dysfunction causes primary arterial hypertension, whereas flow-limiting atherosclerotic lesions lead to acute and chronic ischemic syndromes (e.g., coronary heart disease, myocardial infarction, apoplexy). Stiff heart develops in the form of concentric hypertrophy characterized by pathologic fibrosis resulting in diastolic heart failure, atrial arrhythmias, and degenerative aortic stenosis.

Thus, stiff tissue/organ remodeling leads finally to dyslipidemia, insulin resistance/T2D, and arterial hypertension, the classic components of the so-called metabolic syndrome. The development of these risk factors because of organ stiffening in turn increases endothelial dysfunction, closing another positive feedback loop (Fig. 1).

After all, “equipotent” therapy regimes for risk factors, which concurrently reduce tissue stiffness, are more effective in reducing cardiovascular mortality/morbidity than their “non anti-fibrosing” counterparts:

- For insulin resistance/T2D: SGLT2 inhibitors, biguanides v insulin, sulfonylureas, acarbose
- For hyper-dyslipidemia: CSE-inhibitors v ezetimibe
- For hypertension, heart failure: ACE and ATI inhibitors, aldosterone antagonists v dihydropyridines, alpha inhibitors

## Conclusion

Endothelial stiffening caused by persistent endothelial activation leads via the formation of an inflammatory, stiff ECM, to inflammation and proliferation of the vascular media with consequent wall thickening/stiffening. Reciprocal coupling from the stiff ECM to the surrounding organ tissue triggers fibrosis and epithelial stiffening with increasing organ stiffness and consecutive metabolic alterations. The consequences of this mechano-metabolic coupling are arterial hypertension, dyslipidemia, and insulin resistance/T2D, the so-called metabolic syndrome. This is — contrary to the current doctrine — not the cause, but the consequence of increased vessel/organ stiffness and leads to further endothelial damage constituting a positive feedback loop (Fig. 1). Applying Occam’s razor, there is a single cause, a change in the mechanical properties of tissues to higher stiffness, which leads to a large number of symptoms and organ malfunctions including metabolic syndrome, instead of multiple causes in prevailing doctrines. On the basis of the hypothesis here presented, the therapy of cardiovascular risk factors (hypertension, hyperlipidemia, insulin resistance/T2D) should pay attention first to a decrease in the pathogenetically underlying increased tissue stiffness (e.g., activity reduction of ENaC, RhoA, F-actin, and YAP/TAZ) instead of a meticulous correction of surrogate quantities hypertension, hyperglycemia, and hyperlipidemia.

## Data Availability

Not applicable — This work did not process any raw data but only develops a hypothesis. Data that could be provided to the public do not exist.
